# Uncertain pathogenicity of Pasteurella bettyae in genital and extra-genital sites: insights from a French series of 25 cases

**DOI:** 10.1136/sextrans-2025-056567

**Published:** 2025-07-11

**Authors:** Jean Berset de Vaufleury, Marie Danset, Françoise Truchot, François Durupt, Olivier Dauwalder, Laurie Gouillon, Matthieu Godinot

**Affiliations:** 1 Department of Dermatology, La Croix-Rousse Hospital, Lyon, France; 2 Department of Infectious Diseases and Tropical Medicine, Hospices Civils de Lyon, Lyon, France; 3 Microbiology Platform, Infectious Agent Institute, La Croix-Rousse Hospital, Lyon, France

**Keywords:** Sexual and Gender Minorities, Sexual Behavior, BALANITIS, Homosexuality, Male

## Abstract

*Pasteurella bettyae* is suggested as an emerging sexually transmitted pathogen in men who have sex with men. We analysed 25 cases, revealing a wider distribution and frequent concomitant infections. Our findings challenge its primary pathogenic role, suggesting it may act as an opportunistic coloniser.

*Pasteurella bettyae* is a Gram-negative bacillus sporadically reported in human infections, such as felons,^1^ mostly isolated from urogenital samples^2 3^ particularly in females.^4^ Recent studies suggested that *P. bettyae* may be an emerging sexually transmitted pathogen, especially among men who have sex with men (MSM) in Europe.^5 6^ However, its role as a primary pathogen remains uncertain.

The present retrospective study, conducted in the Hospices Civils de Lyon (HCL), analysed all cases with *P. bettyae* isolated from various samples between January 2019 and August 2024; 2019 was chosen to precede the first published reports (2021) that suggested *P. bettyae* as a possible sexually transmitted infection (STI). Adult patients (≥18 years) with at least one *P. bettyae*-positive sample were included, and samples were categorised as genital, peri-genital (pubis, groin, perineum, gluteal cleft, anal, buttock) or extragenital. All samples were processed manually or using the WASPLab and incubated on the commercial culture medium (bioMérieux/Copan). Identification was performed using MALDI-TOF Vitek MS (bioMérieux). Systematic molecular testing for *Chlamydia trachomatis* (CT) and *Neisseria gonorrhoeae* (NG) was performed for genital samples. Data were anonymised before analysis to comply with ethical guidelines.

*P. bettyae* was identified in 25 cases: 17 genital (68%), 2 peri-genital (8%) and 6 extragenital (24%) samples. Antibiotic therapy was initiated in 17 cases (68%), and symptom resolution or improvement was observed in 52.9% (9/17). Clinical outcomes were available in 17/25 patients.

The genital samples were obtained from 6 females and 11 males, of whom 10 identified as MSM; 6 MSM had balanitis or balanoposthitis and 4 presented with urethral symptoms. The non-MSM male patient had urinary symptoms, and his midstream urine culture was positive for *P. bettyae, Citrobacter koseri* and *Klebsiella aerogenes*. Among female patients, symptoms included leucorrhoea, pruritus and pelvic pain (table 1). Concomitant infections were frequent (10/17), including 3 cases among males with confirmed urethritis pathogens (2 with NG and 1 with CT) and 3 cases among females with confirmed vaginitis or vaginosis pathogens (1 with CT and *Gardnerella vaginalis* (GV), and 2 with GV only). In cases without documented concomitant infections, alternative diagnoses such as histologically proven inflammatory dermatoses were favoured, and symptom resolution was achieved using topical corticosteroids (cases 1 and 6, table 1).

**Table 1:** Clinical and microbiological details of the 17 cases with genital samples *Pasteurella bettyae*-positive

	Sex	Age rank	Year	Sexual orientation	Swab location	Symptoms	Most probable diagnosis	Argument(s) for most probable diagnosis
1	M	50–54	2022	MSM	Glans	Erythema, scales	Psoriasis	Glans histology confirming psoriasis
2	M	50–54	2021	MSM	Glans	Plaque, ulceration	Primary syphilis	Symptoms indicative of syphilis, healed after IM benzathine benzylpenicillin, four other germs on swab (Ct- Ng-)
3	M	20–24	2022	MSM	Glans	Erythema, whitish sludge	Mycosis	Improvement after topical econazole
4	M	30–34	2023	MSM	Glans	Erythema	Bacterial balanitis	Two other germs (Ct- Ng-), lack of response to econazole
5	M	25–29	2024	MSM	Glans	Brown macules	Post-inflammatory pigmentation	Chronic evolution, swab before (Pb+) and after (Pb-) benzathine benzylpenicillin without clinical change, medical history of glans Mpox with lesions at the same location
6	M	50–54	2024	MSM	Glans	Erythema, ulceration	Lichen planus	Glans histology confirming lichen planus
7	M	25–29	2021	MSM	Urethra	Burning when urinating	Ng urethritis	Recent unprotected sexual intercourse with a partner who presented a pharyngeal PCR Ng+ at that time
8	M	20–24	2019	MSM	Urethra	Purulent discharge	Ng urethritis	PCR Ng+ and healed after IM ceftriaxone and doxycycline
9	M	30–34	2019	MSM	Urethra	Clear discharge	Ct urethritis	PCR Ct+ and healed after doxycycline
10	M	40–44	2024	MSM	Urethra	Burning when urinating and discharge (no precision)	Ng urethritis	PCR Ng+ and healed after IM ceftriaxone and doxycycline
11	M	50–54	2024	unk	Midstream urine	Burning when urinating and pelvic pain	Urinary tract infection (faecal germs)	*Citrobacter koseri, Klebsiella aerogenes,* Pb all positives on two midstream urine swab
12	F	25–29	2019	unk	Vagina	Pruritus	Ct infection	Gv on cervical smear and vaginal PCR Ct+
13	F	20–24	2020	unk	Vagina	unk	Gv vaginosis	Gv on vaginal swab
14	F	45–49	2021	unk	Vagina	Pelvic pain, upper genital infection	None	None
15	F	30–34	2023	unk	Vagina	Pain, spotting	Vaginosis	Anaerobic flora and *Klebsiella pneumoniae* on vaginal swab
16	F	35–39	2024	unk	Vagina	Leucorrhoea	Mycosis and Gv vaginosis	Gv and *Candida albicans* on vaginal swab
17	F	18–19	2024	unk	Vagina	Chronic pelvic pain	Multifactorial chronic pain	Chronic and severe pelvic pain with extensive negative investigations combined with psychiatric comorbidities (polyaddiction and complex psychosocial background)

Ct, *Chlamydiae trachomatis*; F, female; Gv, *Gardnerella vaginalis*; IM, intramuscular; M, male; Mpox, Monkeypox; MSM, men who have sex with men; Ng, *Neisseria gonorrhoeae*; Pb, *Pasteurella bettyae*; unk, unknown.

Peri-genital samples were obtained from two MSM with HIV infection. One presented with seborrheic dermatitis, while the other had *Staphylococcus aureus* superinfected eczema (bacteriologically and histologically confirmed), which provided an alternative explanation to their inguinal intertrigo. Extragenital samples were obtained from three females and three males, comprising two cases with post-abdominal surgery with inflammation or discharge, two hand felons, one chronic wound of the foot and one intraoperative nasal sample.

In this series of 25 cases, *P. bettyae* was frequently identified with other microorganisms, including commensals or pathogens. As opposed to previous studies^5 6^ in which *P. bettyae* was associated with balanitis in MSM, our findings suggest a wider distribution among both heterosexual males and females and MSM, with diverse clinical presentations often related to alternative aetiologies. However, the identification of *P. bettyae* in extragenital sites does not preclude sexual transmission; coexisting transmission routes exist and should be considered. The high proportion of MSM in our series likely reflects a detection bias. Overall, males are more likely to undergo STI testing than females.^7^ Additionally, MSM are encouraged by national guidelines to undergo quarterly HIV screening, further increasing their frequency of testing and likelihood of *P. bettyae* identification. While males represent 60–73% of attendees in our sexual clinics, including 34–62% MSM, data regarding the proportion of non-MSM with bacteriological testing are not available.

Notably, an alternative clinical condition was identified in more than half the cases in which *P. bettyae* was the sole microorganism isolated, supporting the hypothesis that it primarily acts as an opportunistic coloniser rather than a causal pathogen.

The major strength herein is the systematic collection of all *P. bettyae*-positive samples across multiple departments within the HCL, providing an accurate epidemiological representation. However, the retrospective nature of the study limits the ability to establish causal inference between *P. bettyae* and clinical symptoms, particularly due to a lack of patient’s history regarding sexual practices and animal contact, since the main reservoir of *Pasteurella* is cats and dogs.

The frequent detection of *P. bettyae* in symptomatic MSM may suggest a role as an STI. However, the presence of co-pathogens and alternative diagnoses in several cases also suggests an opportunistic coloniser behaviour.
